# Influence of the Structure and Mechanisms of Intermetallic Phase Formation on the Strength Properties of a Newly Developed Solder Joint

**DOI:** 10.3390/ma18030489

**Published:** 2025-01-22

**Authors:** Bożena Szczucka-Lasota, Tomasz Węgrzyn, Bogusław Łazarz

**Affiliations:** Faculty of Transport and Aviation Engineering, Silesian University of Technology, Krasinskiego 8, 40-019 Katowice, Poland; tomasz.wegrzyn@polsl.pl (T.W.); boguslaw.lazarz@polsl.pl (B.Ł.)

**Keywords:** soldering, Sn-Pb-Ag-Cu-Al, intermetallic formation mechanism, mechanical resistance, microstructure, alternators, automotive industry

## Abstract

The current development of soldering materials focuses on the properties of the solder itself, while the reliability of the joints formed after soldering is evaluated to a lesser extent. It is essential to understand the relationship between the structure and the strength of the solder joint obtained. This article shows that the properties of the material used for soldering only to a certain extent largely translate into the mechanical properties of the joint. The aim of this article is to emphasize the importance of the problem of the selection of the chemical composition of the solder with the simultaneous selection of the parameters of the soldering process, including the width of the solder gap for the selected strength properties of the connection. The purpose of this article is to emphasize that the selection of a chemical composition solder with a simultaneous selection of parameters of the soldering process, including the dimensions of the gaps of the soldered materials, are affected properties of the soldered joint. In this article, the main importance was focused on the chemical composition of a tin-based solder. The influence was analyzed, and the most favorable content of elements, such as Al and Cu, which create intermetallic phases, strengthening the soldered joint, was determined. The properties of the newly developed solder joint for alternator applications due to the specified conditions of the soldering process, including the width of the solder gap permissible for alternators, ensured the correct connections, the strength properties of which differed despite the use of the same soldering material and substrate material, as well as the soldering time and tip temperature. This article presents a change in the cracking model of a solder joint made using a newly developed material due to the width of the permissible solder gap in the production process of alternators.

## 1. Introduction

To improve the performance of solder joints used in the electronics industry, researchers are developing new solder pastes or fluxes with altered chemical compositions. The modifications are aimed at obtaining the best possible joints in the soldering process for industrial use. A crucial element is to conduct structural studies and assess the impact of the microstructure obtained in the process on the properties of the connection. Based on the theory of diffusion mechanisms occurring in metallurgical processes, it was found that doping soldering alloys with metal particles may contribute to significant changes in the solder structure obtained in the process, characterized by the precipitation of hard intermetallic phases [1,2,3]. It was found that the appropriate arrangement of hard, fine precipitates can affect the dispersion strengthening mechanisms of the structure and, thus, significantly improve the solder matrix and increase the mechanical properties of the solder joints. The influence of alloying element additions on the basic mechanism of formation and growth of intermetallic phases and the width of dendritic bands in the structure of the solder joint may be significant [4,5,6]. The microstructure of a Sn-based eutectic alloy with Pb can be altered by adding a small amount of a given alloying element to the bulk microstructure [7,8,9]. Alloying elements added to the solder shape the solder and the formation and growth of IMC phases, and a given addition can both limit the growth of hard and brittle intermetallic phases and affect their development; the mechanism of formation of a given phase is conditioned by diffusion processes, the initial content of a given element in the solder, as well as the presence of other additives [10,11,12]. The influence of selected elements on the growth of intermetallic phases in the Sn-based solder structure is presented in Table 1. The element content should be considered as a guideline. The values given are consistent with the test results presented in the references. Still, the chemical compositions of the tested solders based on Sn were not always modified with a single additive, which significantly limits the possibilities of comparison and precise determination of the limit up to which the positive effect of a given element on the properties of the obtained solder joint is noted.

In addition to the elements that are deliberately added to the solder, the structure of the forming junction is affected by the diffusion processes between the base material and the solder as a result of diffusion processes, and copper from the substrate material enters the solder joint material. As indicated by the literature data [10], the average thickness of the intermetallic layer for a small volume of solder is more significant than for solders with a large volume of solder. This is because the concentration of Cu in solder grows faster in smaller solder balls than in larger ones. This also means that the IMC layer is highly dependent on the surface finish material during the soldering process, as well as the time and temperature of the process, which determine how significantly surface metallization dissolves in the molten solder. In the case of cold soft solders, this relationship, although smaller than in the case of hot soldering, is still more significant. To reduce or increase the rate of growth/reaction of IMC and to effectively control the formation of an additional reaction layer at the interface between a metal and a solder, it is necessary to carefully select the chemical composition of the solder, as well as the parameters of the soldering process. For conductive connections (e.g., in alternators, electronic systems), novel solutions are sought to reduce the use of harmful lead, as well as to create a durable connection that is stable in operating conditions. On the other hand, the miniaturization of systems should be considered. Information contained in the literature indicates that lead-free connections do not always meet complex requirements. For example, authors Mazullah, Khan et al. investigated the mechanical properties of the Sn-Pb lead soldering alloy and compared it to lead-free solders containing Ag, Cu, Bi, and Zn additives [7]. They found that the expanded IMC phases, which play a significant role in the failure of the solder joint, formed in the lead-free joints they studied, despite the addition of Zn, which has a limiting effect on the growth of intermetallic phases. The published results of the research allow us to understand the Cu_6_Sn_5_ phase, and a beneficial slowdown of the growth of the intermetallic phases is observed in the case of eutectic alloys containing Pb with the addition of alloying elements such as Al, Zn, and Ni.

Adding an appropriate amount of Ag to the soldering alloy can promote the formation of an eutectic lattice structure and a heterogeneous nucleation reaction of β-Sn/Cu_6_Sn_5_ and refine the grain size of β-Sn [38]. This change is due to the formation of Ag_3_Sn in the metal solder diet. Fine precipitations of the Ag_3_Sn phase, distributed evenly in the eutectic volume, slow down the development of the Cu_6_Sn_5_ phase and are beneficial for maintaining good mechanical properties of the connection. It should be noted that according to the results of research presented by Kim et al. [9], a Ag content above 3% promotes the growth of the intermetallic phase, which results in a change in the mechanism of solder cracking. The formation of large Ag_3_Sn inserts causes severe deterioration in the mechanical properties of the joints. Therefore, according to the authors of the study, the Ag content must be below about 3.2% by weight to avoid the formation of large Ag_3_Sn platelets. The crack surface of solder joints with the addition of Ag shows typical ductile cracking, with a Ag content of less than 3.2%, while solders with a high Ag content show a mixture of ductile and brittle cracks. The authors of the publication also showed that shear cracking depends on the presence of large Ag_3_Sn platelets near the interfacial reaction layer. The presence of large Ag_3_Sn plates determines the fracture pattern in tensile and shear tests. The authors found no significant effect of the long whiskers of Cu_6_Sn_5_ on tensile cracking. According to [38], with the addition of Ag, a greater number and a smaller size of IMC particles (Ag_3_Sn and Cu_6_Sn_5_) are observed. In a Sn-0.7Cu-2Ag solder, the number of Ag_3_Sn particles is greater than that of Cu_6_Sn_5_ particles due to the influence of interface energy. Similarly, γ-SnIn4IMC was found in a Sn-0.7Cu-2In solder, which promoted the formation of eutectic areas on the surface of the β-Sn matrix and inhibited the formation of large β-Sn dendritic cells. On the other hand, the addition of the highly active element resulted in the formation of γ-SnIn4 IMC, which reduced the Sn activity and inhibited the formation of Cu_6_Sn_5_ IMC. In lead-free connections, the effect of polarization on the IMC increases on the interface, which was independent of the solder composition. The growth rate of the IMC layer at the anode was always higher than at the cathode. Therefore, the mechanical properties of lead-free solder joints used in electronics may change during operation, with changes related to the increase in ICM phases, caused by the diffusion of ions in the solder material. Wang, F., Li, D., Tian, and others [17] showed that after mixing Sn-Pb with Sn-Ag-Cu, partial or complete mixing clearly reduced both the formation of cracks on the cathode side and the growth of IMC on the anode side. According to a literature review [39,40,41,42,43,44,45,46] scientists improved the strength of solders by alloying, particle strengthening, optimizing the soldering process, and developing matched solder fluxes. However, current development is focused on the properties of the solder itself, while the reliability of the joints formed after soldering is evaluated to a lesser extent. It is essential to understand the relationship between the structure and the strength of the solder joint obtained. The aim of this article is to emphasize the importance of the problem with solder selection and the parameters of the soldering process and their influence on the strength properties of the joints. Due to the selected conditions of the soldering process, the properties of the solder can ensure the correct connections, the strength properties of which will vary and will depend to a greater or lesser extent on the properties of the solder itself and the base material.

## 2. Research Materials

The material used for this study was a solder joint, connecting two copper wires with a cross-section of ϕ1 ϕ = 2 of 1.5 mm, made using a newly developed Sn-1% Cu-0.3%Al-1%Pb-1%Ag solder. The connector was made with the following process parameters:

- Soldered time of 5 s;
- Cave temperature of 380 °C.

The distance between the copper wires in the connectors is between 0.1 and 0.5 mm. The soldering parameters were not changed. The typical soldering parameters were selected using a tin-based solder. This article focuses primarily on the material part, not the technological part of the soldering process.

**Figure 1:**
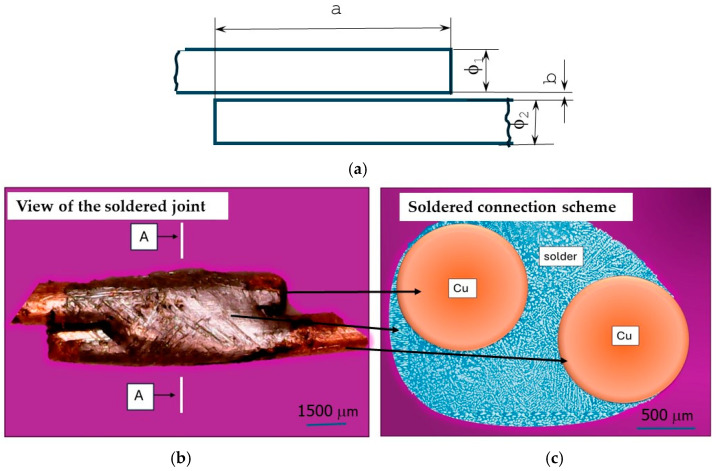
Diagram of solder connection. (**a**) Arrangement of copper wires; (**b**) view of joint; (**c**) diagram of structure connection (cross-section).

The diagram of the solder joint is presented in Figure 1a–c.

According to Figure 1a,b, the copper wires are stacked one above the other in parallel. The solder must penetrate the space between the wires (Figure 1c). The process and the selected material must ensure adequate wettability between the copper substrate and the solder structure produced. No voids should be observed at the border of the separation. A diagram of the predicted eutectic structure of the connection is shown in Figure 1c. The cross-section of the joint is elliptical in shape. The shorter radius of the ellipse is comparable for all tested samples, and the dimension of the radius of the longer ellipse depends on the assumed distance b between the soldered materials and ranges from 1.6 to 1.75 mm. The connection is made on a tab. The length of the joined edge a (Figure 1a) is 1 cm. This is the longest length that could be assumed for the planned application in the construction of the alternator.

## 3. Research Methods

The aim of this study was to determine the relationship between the chemical composition and the structure of the obtained joint and the relationship between the strength in the tensile test of the obtained joint and the microstructure of the solder.

### 3.1. Visual Studies of Macrostructure

After the soldering process, visual tests were performed to assess the quality of the solder joint. Visual examination (VT) of the solder joints was performed using a Reflecta USB200 electronic magnifier(producer Cynel-Unipress Sp. z o.o. Białołęcka 231 B Street, 03-253 Warszawa, Poland) connected to a computer at a magnification of 10×. The tests were conducted in accordance with the requirements of PN-EN ISO 17638 [47] and assessment criteria according to EN ISO 5817 [47].

### 3.2. Radiographic Testing of Solder Joints

Then, it was decided to perform radiographic tests according to the EN 17636-1 standard. Solder joints of two copper wires with a diameter of 1.5 mm, connected over a section of 15 mm, were used for the research. Previously, the wires were mechanically cleaned and moistened with flux FLUX-TKP-101 (producer Cynel-Unipress Sp. z o.o. Białołęcka 231 B Street, 03-253 Warszawa, Poland). The copper wires were placed in a special holder that ensured their stable position over a distance of 10 cm during the examination. Positioning was crucial to obtain a uniform radiographic image and the appropriate detection quality of possible defects. The radiographic equipment was then calibrated, adjusting the exposure parameters to the requirements of EN 17636-1 [47]. The device settings were configured to produce high-quality images equivalent to the W18 quality class.

Visual and X-ray examinations made it possible to assess the quality of the solder joint. The results and their analysis are presented in the next chapter.

### 3.3. Studies of the Microstructure of LM and SEM

To determine the microstructure of the junction, tests were performed using light microscopy (LM) (Zeiss Axio Observer.Z1m; manufacturer: Carl Zeiss Microscopy GmbH, Jena, Germany) and the SEM scanning electron microscope Zeiss Supra 35 (Zeiss Supra 35; manufacturer: Carl Zeiss NTS GmbH, Oberkochen, Germany), with an accelerating voltage of 20 kV and magnifications of 70–15,000×. The investigation was carried out on transverse specimens. With the use of attachments, the chemical compositions of the tested samples were determined, and the occurrence of diverse types of intermetallic phase precipitation and uniformity of the distribution of these phases in complex eutectics and the ß(Sn) phase areas were confirmed. The width of the bands of the interdendritic regions was measured. Analyses of chemical composition in micro-areas were performed using an EDX detector (Thermo Scientific™, EDX detector: Thermo Fisher Scientific, Waltham, MA, USA) with the Pathfinder software (thermofisher.com) (EDX detector: Thermo Fisher Scientific, Waltham, MA, USA). This stage was performed using an EBSD camera (Orientation Imaging Microscopy v5 Analysis software version 5.31) and the OIM Analysis software from EDAX.

### 3.4. Strength Tests

The aim of this study was to check whether the strength of the joint made with the use of the newly developed solder is higher than that of traditional solders used in the production of alternators. The manufacturing methods can affect the properties of the obtained joint. Thus, the properties of the solder stick, as determined by pre-manufacture endurance tests, may not reflect the behavior and strength properties of the joint at the end of production. The joint is characterized by a specific structure, and during the test, there is a complex state of stress in it. There is cooperation between the substrate material and the solder, so its strength will depend relatively on many factors, e.g., wettability, adhesion, and microstructure, and it is a complex system. Therefore, this article compares the results of endurance tests obtained for the new connector with the results of tests obtained for traditional, previously used soldered joints in alternators. Due to the lack of ASTM guidelines for testing the strength of solder flaps with very small cross-sections (the diameter of the wires to be joined is 1.5 mm), the authors of this article decided to carry out the test based on the ASME Section IX: Welding, Brazing, and Fusing Qualifications guidelines for soldering joints, as the joints are most similar to those presented in this article. Each tensile test consisted of gradually increasing the load until the joint broke. The test is, therefore, for reference only and indicates whether the tested joint is characterized by increased strength concerning other joints assessed under the same conditions and whether the joint made using the newly developed solder shows the same strength for all samples, regardless of the adopted solder gap. The calculated resistance of the joint to the complex state of tensile and shear stresses is an estimate in a certain range. The test was conducted using the INSTRON 3369 testing machine.

## 4. Research Results and Their Analysis

### 4.1. Results of Macroscopic and X-Ray Examinations and Their Analysis

**Figure 2:**
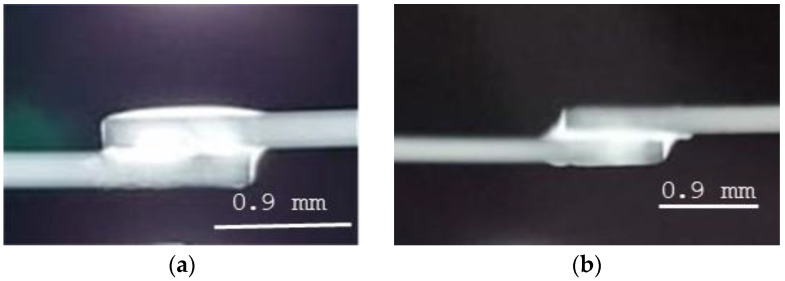
X-ray results of a solder joint (**a**) with a solder gap of (**a**) 0.1 mm and (**b**) 0.3 mm.

As preliminary examinations, visual and X-ray examinations of the soldered joints were carried out, which were aimed at assessing the correctness of the connections. Macroscopic observation of the solder joints allows us to conclude that the joint is uniform, and the solder has been evenly distributed on the surface of the joined elements. Tests did not identify gaps, voids, or areas where the solder did not adhere to the base material. Macroscopic evaluation of the solder confirmed that in all samples, the solder is smooth, shiny, and homogeneous, which indicates that the joint was properly made. The results of the X-ray examinations confirmed that the base material was properly melted with the solder (Figure 2a,b).

Proper penetration is a guarantee of good adhesion and strength of the joint in mechanical tests.

The results of macroscopic and X-ray examinations are presented in Table 2.

During the tests, no surface defects were detected, such as porosity, air bubbles, micro-cracks, pitting, or other surface discontinuities that could affect the mechanical strength of the joint (Table 1). The X-ray examination also did not identify air bubbles or pores in the solder that could indicate contamination, improper flux, or too rapid cooling of the joint (Figure 2). All connections showed the correct structure.

### 4.2. Microscopic Results and Their Analysis

**Figure 3:**
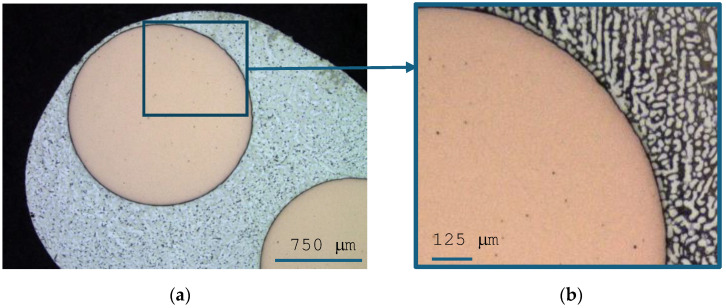
Joint made using the Sn-1% Cu-0.3%Al-1%Pb-1%Ag solder. (**a**) General view; (**b**) connection between solder and copper surface.

The results of the LM observation of the microstructure of the joint obtained with the use of the Sn-1% Cu-0.3%Al-1%Pb-1%Ag solder, for which the solder gap was 300 μm, are presented in Figure 3.

**Figure 4:**
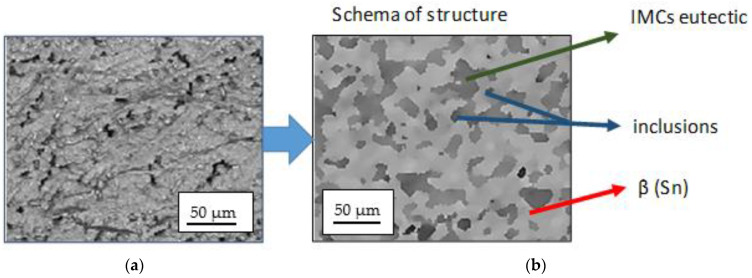
The joint made using the Sn-1% Cu-0.3Al-1%Pb-1%Ag solder. (**a**) Solder microstructure; (**b**) structure diagram.

We can clearly see that the Cu substrates were well wetted by the solder alloys during soldering, and no visible voids were observed in the solder joints. The result confirms that the connector was made correctly. Eutectic areas can be observed in the junction structure. The most important properties of a solder, affecting the strength of the solder joint in alternators, result from the joint structure formed in the soldering process. Essential elements of the structure determining the properties of a solder are intermetallic and micro-structural formations formed in the solder. Both their distribution and size determine the strength properties of the connection. Figure 4 shows a solder joint, obtained using a Sn-1%Ag-1%Pb-1%Cu-0.3%Al solder, with clearly visible inclusions.

**Figure 5:**
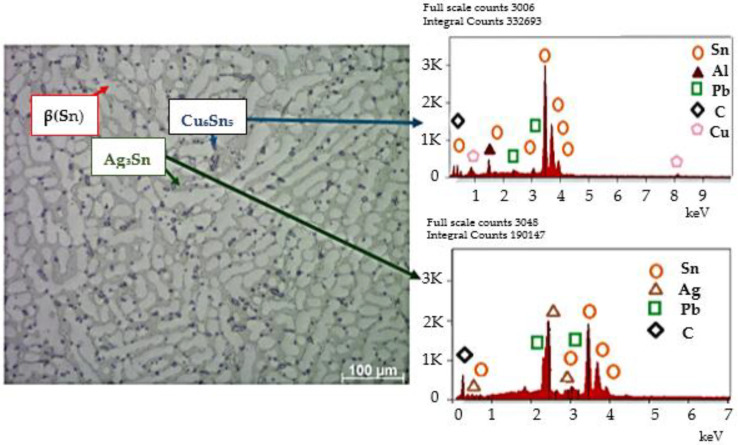
Schema of microstructure of SEM solder with analysis of the chemical composition of precipitates.

**Figure 6:**
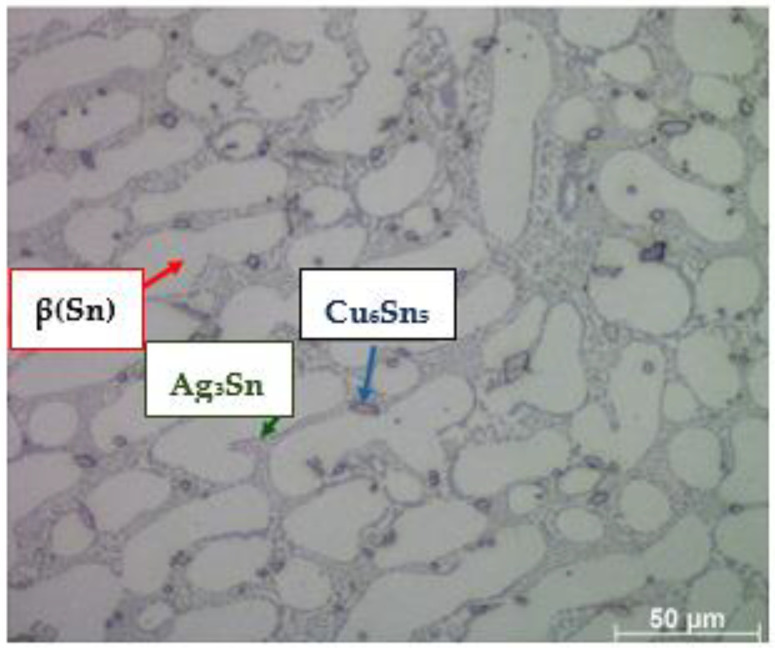
Microstructure of SEM solder with analysis of the chemical composition of precipitates.

The chemical composition of the newly developed solder, containing Sn-1%, Cu-1%, Al-0.3%, and Pb-1%Ag, allowed for obtaining a structure characterized by the presence of β(Sn) phase areas (light gray areas in Figure 4), an eutectic composite matrix (dark gray areas in Figure 4), as well as numerous inclusions, both in the matrix and slightly more extensive ones, most often located along the phase boundaries (very dark areas in Figure 4). As shown in the literature, the presence of metal particles such as Ag or Cu in solder alloys changes the structure of the solder matrix and enables the formation of new compounds through metallurgical reactions occurring in the soldering process [16,17,18]. On the one hand, the resulting precipitates (inclusions) slow down the diffusion processes, and on the other hand, they strengthen the strength of the solder [11,12,13]. The literature analysis also confirms that the addition of the analyzed metal particles has a negligible effect on the melting point but can significantly enhance the mechanical properties of soldering alloys. The obtained structure (Figure 1 and Figure 2) confirms the formation of small inclusions both at the grain boundary and inside the eutectic structure. The joint has been made correctly; it is characterized by the homogeneity of the structure in volume. The analysis of the microstructure of the junction using a scanning microscope, together with an X-ray microanalysis of the chemical composition, allows for the conclusion that the intermetallic phases are formed in the eutectic matrix of the β-Sn type (dark points in Figure 5 and Figure 6).

These phases are crucial for the properties of the solder joint. The microscopic observation (Figure 6) allows us to determine the following:

- Even distribution of the intermetallic phases in the eutectic matrix;
- The areas of the intermetallic phases do not show excessive growth.

Such a formation of intermetallic phases in the soldering process ensures obtaining joints with appropriate strength properties, which is confirmed by the literature data [12]. The material, consisting of an eutectic matrix and reinforced with the evolved fine intermetallic phases, has the properties of a particle-reinforced composite. It should be emphasized that research and publications in this field are a scientific novelty and are not extensive. Available information in the scientific literature confirms that most researchers have noticed that fine hard phases evolved in the matrix or introduced intentionally into the solder improve the properties of the solder joint, such as wettability and mechanical properties.

The formation of Cu_6_Sn_5_ and Ag_3_Sn phases and the increase in the β-Sn phase are, therefore, crucial for the quality of the joints obtained. According to the literature data [2,3], the evaluation of solder joints should include the control of intermetallic formations. The growth of Ag_3_Sn and Cu_6_Sn_5_ nanoparticles is interrelated. In the case of the nanoparticles of the intermetallic phase aggregate on the surface of IMCs, they hinder the diffusion of Sn and Cu ions and, thus, slow down the growth of the Cu_6_Sn_5_ layer. The microstructure of the solder, together with the analysis of the chemical composition of the precipitates, is presented in Figure 5.

Figure 6 enlarges the area of intermetallic phases in the composite matrix. The gray areas in Figure 6 are complex IMC (intermetallic composite) eutectics with fine precipitates of the Ag_3_Sn phase. Very bright areas with elongated, often dendritic shapes are the β phase (Sn). Smaller areas with a distinct dark envelope are the Cu_6_Sn_5_ phases. No voids were identified in the analyzed structure.

The results obtained are consistent with the observations made by A.A. El Daly [39], who hypothesized that the higher solidification velocities of β-Sn limit the growth time of the intermetallic phases of Ag_3_Sn and Cu_6_Sn_5_. A similar mechanism has also been described by Li, Y et al. The researchers added graphene to the Sn-Ag-Cu soldering alloy. The addition of graphene increased the thermodynamic resistance of IMC growth and, thus, suppressed the growth of the intermetallic phases [40].

**Figure 7:**
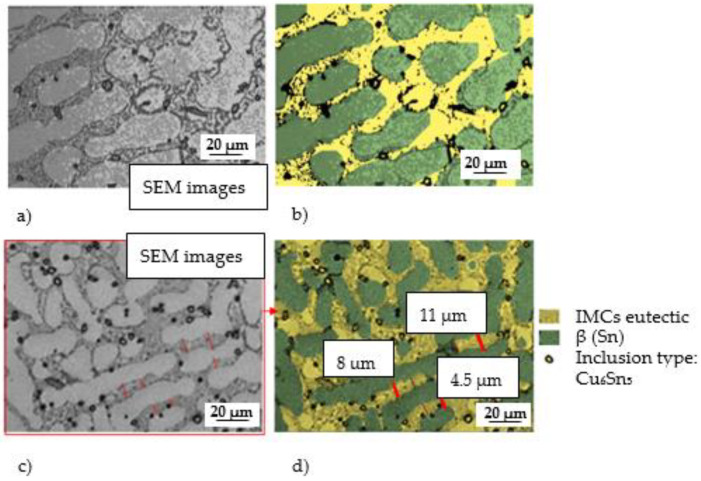
SEM microstructure and solder diagram (**a**,**b**) with a solder gap of 0.4 mm and (**c**,**d**) with a solder gap of 0.1 mm of structure.

The researchers showed that the intermetal-reinforced lead-free composite they studied had better mechanical strength than the lead solder. Therefore, it can be assumed that the observed unexpanded, uniformly distributed intermetallic phases in eutectics will have a strengthening effect (Figure 7). The hypothesis will be verified with tensile strength tests. In addition, the facts quoted from the literature indicate that the introduction of a small amount of an additional ceramic element reduces diffusion processes by increasing the thermodynamic resistance of IMC growth.

Zhang et al. [41] found that the doping solder (containing Cu, Sn, and Ag) with the addition of aluminum affects the formation of Cu_6_Sn_5_ and Ag_3_Sn IMC grains and the movement of dislocation, as well as it determines the structure of the matrix obtained in the soldering process. In the studies presented by the authors, the average distance between dendrites in the matrix of 15 μm (in the absence of Al in the solder) decreased to 5 μm when 0.1 wt.%. Al nanoparticles were incorporated into the solder. Similar observations were made by [13,15], who found that the size of the Ag_3_Sn IMC grains depends on the weight content of Al in the solder. With a lower Al content (less than 0.5%), larger intermetallic phase grains are formed, but the primary β-Sn dendrites also grow, and the distance between dendrites becomes smaller, while the addition of higher Al nanoparticle content inhibits the growth of Ag_3_Sn IMC grains. Microstructure studies (Figure 7) confirmed the positive effect of the doping 0.05% Al solder.

**Figure 8:**
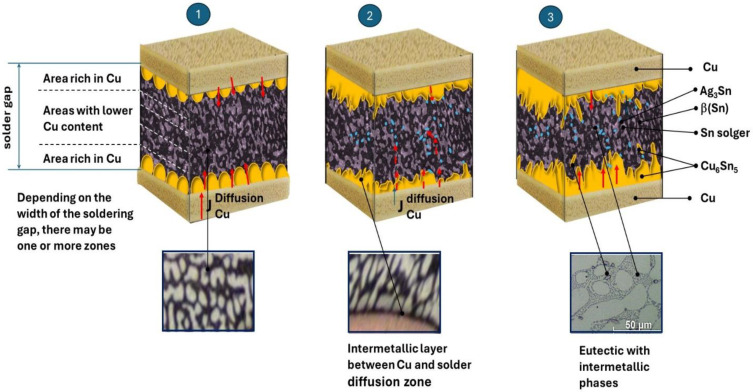
Model of growing the intermetallic phases: (step 1) formation of the eutectic structure and the beginning of the formation of the intermetallic layer; (step 2) chemical reactions in the solder, formation of very fine precipitates of the Ag_3_Sn phase, and blocking the diffusion of Cu from the substrate; (step 3) inhibition of the growth of the intermetallic layer at the separation boundary, concentration of the number of fine precipitates of the Ag_3_Sn phase in eutectics, formation of fine, uncoagulated secretions of Cu_3_Sn_5_ phases in eutectics as the second strengthening phase, and inhibition of Ag_3_Sn phase growth. Red arrows indicate the direction of the diffusion of elements.

Inhibition of the growth of the intermetallic phase affects the diffusion processes occurring within eutectics and promotes the enlargement of dendritic areas at the expense of the growth of the intermetallic phase. Thus, the doping of the Al solder affected not only the growth of IMC but also the mechanical performance of the resulting junction. Inhibition or significant slowing down of the formation of brittle, expanded intermetallic phases is a desirable phenomenon. It is known that the thickness of the IMC layer at the interface between the solder and the Cu substrate is crucial for the reliability of soldered joints. In particular, the thickness of the IMC layer affects the reliability of connections during long-term operation or accidental dropping of the soldered element (during production, assembly, etc.). Therefore, the dissolution of Cu from the copper substrate must also be tightly controlled in industrial products to allow for the formation of an IMC layer with the desired thickness at the interface. According to the model of the growth of intermetallic phases (Figure 8), the resulting eutectic structure slows down the processes of diffusion of copper from the substrate.

The initially formed Cu_6_Sn_5_ layer at the interface between the substrate material and the solder is inhibited by both the eutectic structure and the exceptionally fine phases of Ag_3_Sn, which bind Sn and limit the diffusion processes. The film formation is very narrow (Figure 8, step 2) when using a newly developed solder. The resulting eutectic structure of Sn-Pb with undeveloped IMC regions is consistent with the observations presented in the literature. It slows down the diffusion processes (Figure 8, step 1). Lee et al. [33] found that the thickness of the IMC layer at the interface of Sn-Ag-Cu solders (solders without lead addition) is drastically higher than in the case of Sn-Pb eutectic solders.

Therefore, the combination of the chemical composition of the solder (Sn-1% Cu-0.3% Al-1% Pb-1%Ag) in the copper joining process is desirable. It ensures the formation of the correct structure of the joint with the following areas:

- Eutectics with intermetallic phases of the Ag_3_Sn type, in which eutectics create a diffusion barrier that prevents a rapid reaction between the applied solder and the Cu substrate, and the Ag_3_Sn particles limit the diffusion of Sn, determining the excessive growth of Cu_6_Sn5 (Figure 8).
- Small particles of the forming Cu_6_Sn_5_ intermetallic phase, which, as the second reinforcing phase, retain their properties unchanged during operation, thus strengthening the soldering alloys, which is confirmed by the literature data [10,11,12,13].
- β(Sn) phase areas.

Studies of the structure of the solder joint allowed for the determination of the role and influence of individual admixtures in the solder composition on the formation of the joint structures in the newly developed solder (Table 3).

To confirm the effect of the obtained structure on the good mechanical properties of the joint, tensile tests were performed.

### 4.3. Strength Tests—Results and Their Analysis

**Figure 9:**
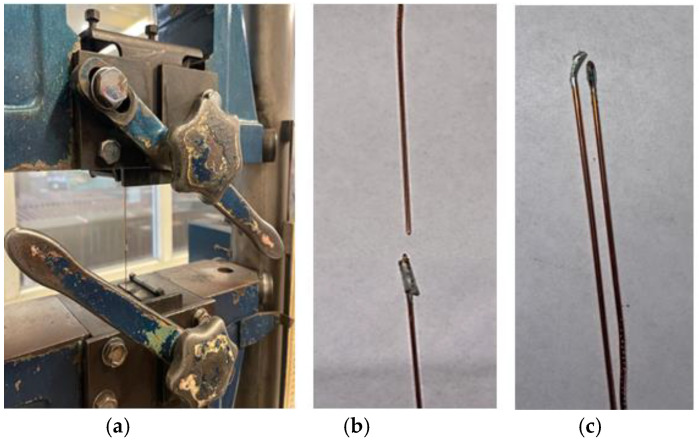
Tensile testing of solder joints. (**a**) Specimen fixation; (**b**) specimen break outside the joint; (**c**) specimen break in the joint.

Stretching of the specimens was conducted on an INSTRON 3369 machine. The specimens cracked both in and out of the junction area (Figure 9).

The results obtained indicate the correctness of the soldered joints made. The tensile and shear strength of the newly developed solder joint was estimated to be above 140 MPa (Table 4 and Table 5).

This is a positive result because joints soldered with a solder based on tin alloys used so far have worse mechanical properties, which were estimated at 64–67 MPa in the test. The results obtained for the connectors used so far are consistent with the literature data [42], so the adopted research methodology can be considered correct. The results confirm that the solder joints obtained with the newly developed solder have 1.5 to 2 times higher tensile strength than the classically used tin alloy joints. Such good properties of the joint are influenced by many factors, mainly related to the structure of the obtained solder, wettability of the solder during the joint manufacturing process, width of the solder gap, behavior of the joint under tensile and shear forces, cohesive forces between the solder and the base material, and correctness of the produced joint.

**Figure 10:**
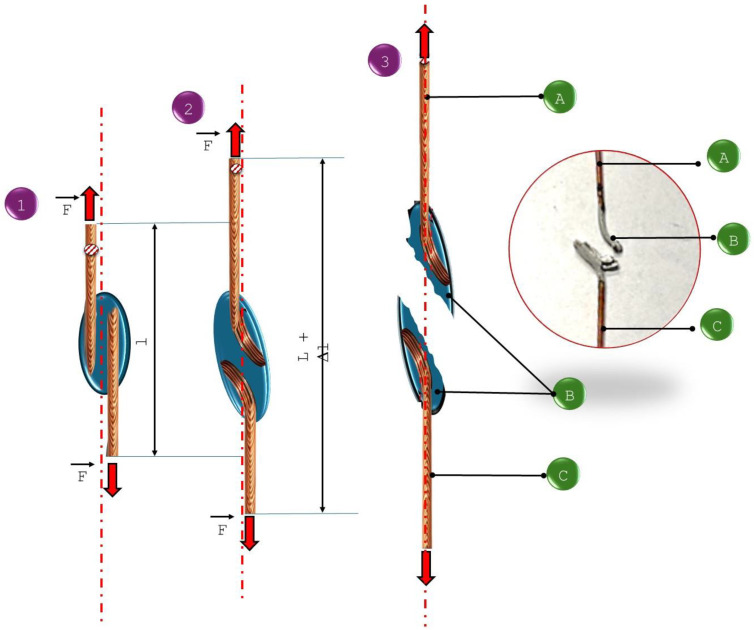
Crack mechanism of a solder with a solder gap below 0.3 mm. A—copper wire (first material); B—solder and C—copper wire (second material).

**Figure 11:**
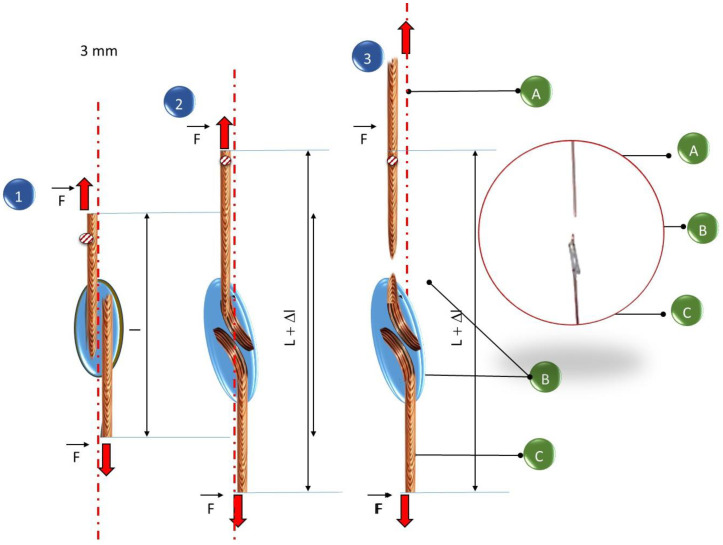
Mechanism of solder joint cracking (with a solder gap above 0.3 mm). A—copper wire (first material); B—solder and C—copper wire (second material).

The tested specimens made using the newly developed solder for the solder gap in the range of 0.1–0.3 mm cracked during the tensile test outside the area of the solder joint as a result of shear forces and deformation of the base material during the test, which indicates high strength of the joint and its correct workmanship (Figure 9). For newly developed joints made with a solder gap of 0.3–0.5 mm, cracks were noted in the joint, and the recorded breaking force was much higher than the force needed to break a classic junction made using a tin–lead solder (Figure 10). Schematically, the mechanisms of cracking depending on the adopted solder gap are shown in Figure 10 and Figure 11. According to the diagrams, tensile forces act on the joint, causing the cross-section of the specimen to lengthen and narrow, as well as shear forces causing the characteristic deformation of the material at the ends of the welded wires. When a wider solder gap was used, the material cracked diagonally in the solder according to the action of shear forces. Cracks in the base material, outside the joint area, in its immediate vicinity, were noted for joints characterized by a narrow solder gap. These cracks were also associated with the simultaneous action of tensile and shear forces, which caused significant deformation of the base material. As a result of bending the base material (copper wire) at the point of contact with the lap joint, the structure of the base material was weakened, which was destroyed under the action of forces.

The analysis of the influence of the width of the solder gap—a critical parameter of the soldering process—on the strength of joints indicates that this relationship is relatively strong because it determines the cracking mechanism, which translates into the acting force needed to break the sample (Table 2, Figure 10 and Figure 11).

**Figure 12:**
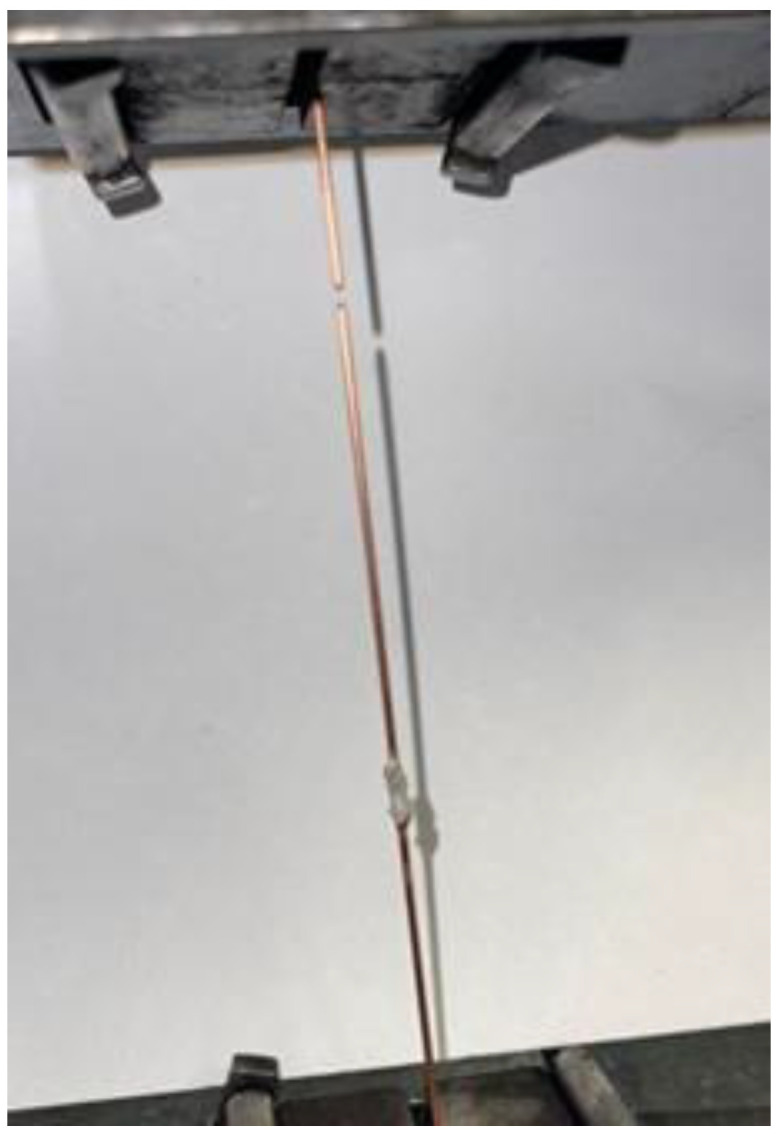
Tensile strength test—fracture of base material.

The results of the shear test of the joints clearly indicate the deterioration of their mechanical properties with the increase in the width of the solder gap. The joint with the smallest gap—0.1 mm—broke under a load of 0.6 kN, corresponding to a shear strength of Rt = 158.3 MPa, and a joint with a solder gap width of 0.5 mm broke with a load of 0.55 kN, corresponding to a shear strength of Rt = 130.95 MPa. The obtained results and their analysis in combination with the crack model (Figure 10 and Figure 11) allow for the conclusion of the following:

- In the case of a narrower joint, the mechanical properties of the materials and the cohesive forces acting affect the strength.
- In the case of wider solder joints, the impact on the shear strength of the material is determined by the shaped microstructure of the solder and the occurrence and distribution of intermetallic phases in it. The results of the research are consistent with the observations made by various authors regarding solders with different chemical compositions than the one in question. For example, in [43], it was found that in joints with a larger gap in the solder microstructure, there is an ASZ zone, the relative volume of which increases with the width of the gap, and a value of >0.3 mm constitutes the entire volume of the solder zone, determining its shear strength properties. Similar relationships were observed in Inconel 718/Palnicro 36M solder joints. The single-phase microstructure of the solder was obtained only in the gap with a width of less than 0.05 mm, while its increase resulted in an increase in the relative volume of the ASZ zone. The publication found that in joints with a solder gap greater than 0.05 mm, the degree of impact of the joint’s microstructure on its strength properties increases with the increase in the width of the joint. In such joints, the most essential thing is to select the chemical composition of the solder. Due to the technological process of alternator manufacturing, the mutual pressing of the copper wires to be joined, without deformation of these materials, can be obtained in the range of 0.1–0.2 mm, and the attempt to obtain a narrower solder gap was not repeatable for the production process. Therefore, the analysis of the obtained research results and their comparison with the literature data allows for the conclusion that the good strength properties of the obtained joint are significantly influenced by the correctly selected chemical composition of the solder, which provided a quasi-composite structure with intermetallic precipitates of the Sn_6_Cu_5_ and Ag_3_Sn types in the matrix of complex five-component eutectics with the presence of a proeutectric ß(Sn) phase, which was confirmed by structural studies. Studies have confirmed that intermetallic phases play a significant role in the formation of a mechanical bond between the solder and the base metal, similarly to tin-based solders with gold addition [44,45,46]. As shown in the previous chapter, a prerequisite for the correct combination of solder and the base metal is the mutual diffusion of atoms of the joined phases, which results in, for example, the formation of intermetallic phases in eutectics. In the case of intermetallic phases, covalent bonds are also present between atoms in addition to metallic bonds, which makes intermetallic phases more brittle than pure metals. These phases exhibit the characteristics of brittle materials with a small elastic range, and their presence in the form of overgrown areas in the solder joint can significantly reduce its fatigue strength, especially when the intermetallic phase occurs in the form of a uniform layer at the interface between the scolded metal and the solder. However, if the intermetallic phases in the form of grains are distributed evenly in the joint volume, as was observed during the study of the microstructure of the newly developed soldered joints, they strengthen the joint and increase its mechanical strength. The properties of the microstructure translate into the strength properties of the joint. Hence, the high strengths of joints made using the newly developed solder are recorded in relation to joints made using previously used materials. The thickness of the layers, shape, and variety of the intermetallic phases formed during the soldering process depend mainly on the chemical composition of the materials to be joined, the residence time of the solder joint above the liquidus temperature, and the speed of cooling of the joint immediately after the soldering process, as well as the solder components limiting the excessive growth of brittle phases. The strength of the tested solder joint is influenced by the shape, form, and size of non-metallic inclusions, the excessive growth of which was limited by the addition of Al. Non-metallic inclusions strengthened the matrix with an effect like the reinforcement mechanism occurring in composite materials. It should be noted that the strength of the joint is also affected by good wettability and adhesion of the solder to the copper elements to be joined, which partially contribute to the transfer of tensile stresses. Differences in the strength of joints made of the same solder are the result of a larger width of the solder gap between the materials to be joined. When testing samples with a solder gap width in the range of 0.3–0.5 mm, each time it was found that the sample broke in the solder. The observed oblique crack was the cause of the simultaneous action of shear and tension forces occurring during the test. It should be noted that no detachments or cracks were noticed at the interface between the solder and the substrate material. During the research, a case of cracking of the native material was also noted at a considerable distance from the joint made. The tested specimen achieved the lowest tensile strength before breaking, so the joint should be considered to meet the strength requirements. A crack in the copper wire outside the joint zone indicates a decent quality of the solder joint, and, on the other hand, it indicates a material defect in the wire (Figure 12).

**Figure 13:**
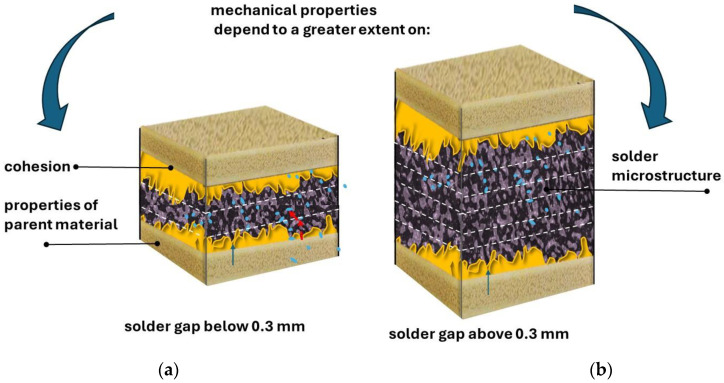
Mechanical properties determined as a function of the properties of the material parent, cohesion, and solder microstructure for a solder gap (**a**) below 0.3 mm and (**b**) above 0.3 mm. Red arrows indicate the direction of the diffusion of elements.

The other results do not indicate that the solder joint has a higher strength than the copper wire, so the real value of the force needed to break was not taken into account in this case when calculating the strength of the connection, and the measurement and result were treated as an error. Diffusion mechanisms conducive to the formation of fine intermetallic phases of the Ag_3_Sn type and mechanisms inhibiting the diffusion of elements causing excessive growth of the Cu_6_Sn_5_ and Ag_3_Sn phases are repeatable in the soldering process (Figure 13). With the established boundary conditions of the soldering process, they enable the creation of the desired joint structure, characterized by good mechanical properties. The process produced a microstructure characterized by fine, evenly distributed hard intermetallic phases in an eutectic matrix. Strength tests confirmed the better properties of joints made using the new solder compared to the currently used connectors. Due to the fact that with a smaller gap width of 0.1–0.3 mm, the mechanical properties of the bonded material and the cohesive forces between the joint and the base material played a greater role in the mechanical tests than the structure of the soldering material, and because the samples showed higher strength, the pressure between the joined components was applied during production, and the gap between the welding wires is now controlled.

**Figure 14:**
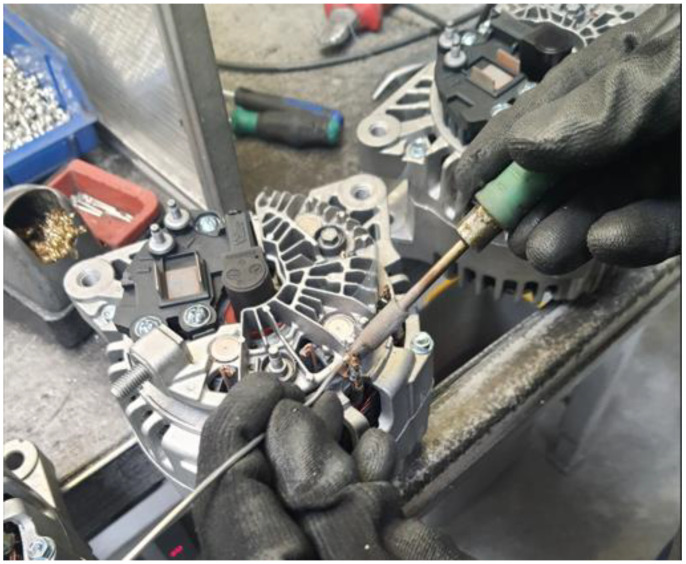
Application of newly developed solder in the production of alternators.

The properties of the joint are determined by both the mechanical properties of the base material and the structure of the solder. The newly developed soldering material was implemented into production (Figure 14).

## 5. Summary and Conclusions

This article presents a soldering method with a newly developed solder based on tin, containing mainly Cu, Ag, and Al additives. Copper and silver are important solder additives, as they contribute to the formation of intermetallic phases that strengthen the joint. In turn, the addition of aluminum affects the limitation of grain growth. Such a selected chemical composition of the solder simultaneously ensures good soldering properties and good mechanical properties of the joint.

The results of structural tests confirm that the soldering process was performed correctly, and the selected chemical composition of the solder (Table 1) and process parameters made it possible to reduce the diffusion of elements, resulting in the formation of the dendritic phase of β(Sn), the formation of intermetallic phases of a reinforcing nature (diffusion strengthening), limiting nucleation and excessive, undesirable growth of the intermetallic phases, and limiting the thickness of interdendritic areas. This structure translated into good mechanical properties of the connection, which was confirmed by the results of a tensile test. As a result of the research conducted, the following were found:

1. Depending on the width of the solder gap, the mechanical properties of the obtained joint depend mainly on the structure of the solder (with solder gaps above 0.3 mm) or are resultant of the mechanical properties of the base material and the solder (with solder gaps in the range of 0.1–0.3 mm). The smaller the solder gap, the more important the cohesive forces between the base material and the solder and the mechanical properties of the base material are for the mechanical properties of the joint.
2. The recorded crack occurs directly in the vicinity of the solder. The wider the gap, the more the structure of the material, including the amount and distribution of intermetallic phases, becomes of major importance.
3. The loss of consistency in the tensile test occurs in the solder material. Solder slant cracking is the result of the simultaneous action of shear and tensile forces during the test.
4. The same chemical composition of solder used to join the base material does not guarantee the same mechanical properties of the joint each time; an essential element is to maintain the stability and repeatability of the process during production and to carefully select the width of the solder gap.

The carefully selected alloy additives for the solder ensure that the desired fine, evenly distributed intermetallic phases in the solder structure are obtained, and the resulting structure has properties like those of a particle-reinforced composite.

It should be emphasized, however, that the area of research on composite-reinforced solders with intermetallic phases is still quite a significant research gap. The effects of the strengthening mechanism observed by researchers are not strongly proven and well documented in the scientific literature, and the issue is the subject of current research.

The last case analyzed during the tests was the crack of the copper wire outside the joint zone, which, on the one hand, indicates a decent quality of the solder joint, and, on the other hand, it indicates a material defect in the wire. In the force measurement, a value higher than the minimum was obtained. The presented investigation was carried out at room temperature. Future tests will include tests at elevated temperatures that correspond to the operating conditions of a car alternator. Thank you for your suggestion regarding the possibility of checking the behavior of the joint in the creep test. We will be interested in conducting research in this direction.

## Figures and Tables

**Table 1:** Influence of alloying elements on the formation of intermetallic phases.

Element	Influence on the Formation of Intermetallic Phases	Ref.
Connection below 0.5% Al	Decrease in the thickness of IMC.	[13,14,15]
Connection over 0.5% Al	Increase in the thickness of IMC; a special phase of the Cu_6_Sn_5_	[13,14,15]
Connection below 3% Ag	Decrease in the thickness of IMC; a special phase of Cu_6_Sn_5_; creation of small phases of Ag_3_Sn.	[9,16,17,18]
Connection over 3% Ag	Creation of overly developed phases of Ag_3_Sn;	
Bi < 3 wt.%.	unchanged ICM.Increase in tensile strength, creep, and fatigue properties. Increase in brittleness and prone to thermal fatigue.	[19,20]
Co	Decrease.	[21]
Cu	Increase in the thickness of IMC; a special phase of Cu_6_Sn_5_ and Cu_3_Sn_5_.	[22,23]
Fe	Unchanged.	[23,24]
Ga	Decrease or Increase.	[25,26]
In	Unchanged.	[13,24,27]
	Increase in the thickness of IMC; a special phase of Cu_6_Sn_5_, but it builds γ-SnIn4 IMC. Decrease in melting temperature. Improved wettability. High ductility and low strength in the case of high In-containing solder alloys. Accelerated oxidation during melting.	
La	Decrease.	[28]
Mo	Decrease.	[29]
Ni	Suppressed growth of the brittle Cu_3_Sn IMC layer. Decrease.	[25,30,31]
P	Increase.	[25,32]
Sb	Unchanged. Forms new IMC Cu_6_(Sn, Sb)_5_ and Ag_3_(Sn, Sb) in the microstructure.	[33]
Ti < 1 wt. %	Forms the new Ti_2_Sn_3_ IMC, which is hard and stiff. Increase in mechanical strength in low weight percentages. Below 1%. Suppressed void formation and coalescence at the Cu (substrate)/Cu_3_Sn interface.	[34,35]
Zn	Decreased but facilitated formation of new IMC (Cu, Zn)_6_Sn_5_ in the reaction layer at the interface.	[36,37]

**Table 2:** X-ray results.

Sample:	Measured Solder Gap b	Tab Length	Macroscopic Examination Result /X-Ray Analysis
L1	0.2 mm	1.1 cm	No solder defects
L2	0.3 mm	0.9 cm	No solder defects
L3	0.3 mm	0.9 cm	No solder defects
L4	0.4 mm	1.0 cm	No solder defects
L5	0.5 mm	1.1 cm	No solder defects

**Table 3:** Chemical composition of the solder and properties of the joint.

Admixture Used	Effect
0.3% Al	Doping of Al solder is justified because it effectively counteracts the beading of IMC, thus positively affecting the mechanical properties of the joint, slowing down the degradation of mechanical properties, and increasing impact resistance. The result is confirmed by measurements of interdendritic areas.
1% Pb	Lead enables the production of eutectics, which is an effective diffusion barrier, preventing a rapid reaction between the applied solder and the Cu substrate. The result is confirmed by structural obstructions of eutectic areas without Cu precipitates and small Cu precipitates recorded at the σ(Sn) interfaces.
1% Ag	Ag combines with Sn and is released on the surface of eutectics, forming the first hard intermetallic phase and a diffusion barrier for Sn, thus limiting the excessive growth of the brittle and hard phase of Cu_6_Sn_5_.
Ok.1% Cu (Cu comes from solder and substrate)	Formation of the Cu_6_Sn_5_ intermetallic phase of a strengthening nature. With the action of mechanisms limiting the diffusion of Sn in metallurgical processes, an unexpanded Cu6Sn5 phase is formed, and the phase forms strengthening inclusions. Diffusion amplification mechanism. Results confirm structural testing in combination with mechanical testing.

**Table 4:** Strength of newly developed Sn-1% Cu-0.3% Al-1% Pb-1%Ag joints in the tensile test.

Sample	Solder Gap [mm]	Ellipse Radius r_1_	Ellipse Radius r_2_	Area of Cross-Section A [mm^2^]	Measured Force [N]	Calculated Strength [MPa]
Sample 1	0.1	0.75	1.61	3.792	600	158.25
Sample 2	0.2	0.75	1.64	3.862	580	150.17
Sample 3	0.3	0.76	1.66	3.961	570	143.89
Sample 4	0.4	0.76	1.72	4.105	580	141.30
Sample 5	0.5	0.76	1.76	4.200	550	130.95

**Table 5:** Tensile strength of 40Sn-60% Pb solder joints used so far (comparative tests).

Sample	Solder Gap [mm]	Ellipse Radius r_1_ [mm]	Ellipse Radius r_2_ [mm]	Area of Cross-Section A [mm^2^]	Measured Force [N]	Calculated Strength [MPa]
Sample A	0.4	0.76	1.73	4.128	280	67.82
Sample B	0.2	0.76	1.63	3.890	250	64.27

## Data Availability

The original contributions presented in the study are included in the article, further inquiries can be directed to the corresponding authors.
